# Thermography and Sonic Anemometry to Analyze Air Heaters in Mediterranean Greenhouses

**DOI:** 10.3390/s121013852

**Published:** 2012-10-16

**Authors:** Alejandro López, Diego L. Valera, Francisco Molina-Aiz, Araceli Peña

**Affiliations:** Department of Engineering, University of Almería, Ctra. Sacramento s/n, Almería 04120, Spain; E-Mails: alexlopez@ual.es (A.L.); fmolina@ual.es (F.M.-A.); apfernan@ual.es (A.P.)

**Keywords:** sonic anemometry, greenhouse, air heaters

## Abstract

The present work has developed a methodology based on thermography and sonic anemometry for studying the microclimate in Mediterranean greenhouses equipped with air heaters and polyethylene distribution ducts to distribute the warm air. Sonic anemometry allows us to identify the airflow pattern generated by the heaters and to analyze the temperature distribution inside the greenhouse, while thermography provides accurate crop temperature data. Air distribution by means of perforated polyethylene ducts at ground level, widely used in Mediterranean-type greenhouses, can generate heterogeneous temperature distributions inside the greenhouse when the system is not correctly designed. The system analyzed in this work used a polyethylene duct with a row of hot air outlet holes (all of equal diameter) that expel warm air toward the ground to avoid plant damage. We have observed that this design (the most widely used in Almería's greenhouses) produces stagnation of hot air in the highest part of the structure, reducing the heating of the crop zone. Using 88 kW heating power (146.7 W·m^−2^) the temperature inside the greenhouse is maintained 7.2 to 11.2 °C above the outside temperature. The crop temperature (17.6 to 19.9 °C) was maintained above the minimum recommended value of 10 °C.

## 1. Introduction

In the Mediterranean Basin, and specifically in the province of Almería (Spain), greenhouses tend to be low-cost structures incorporating few technological climate control methods. While these structures allow suitable conditions for crop development to be maintained during most of the year, extreme heat or cold conditions can lead to crop damage and therefore economic losses. The problem of low temperatures during winter can be solved by supplying some heat to the greenhouse during the critical periods [1,2]. Conventionally, thermal energy is transferred to the greenhouse either by circulation of hot water through a piping system or by air heaters [3,4]. Pipe heating is an effective means of maintaining an appropriate temperature for crops, both by convectively heating the greenhouse air and by radiating heat directly to the leaves, and it is the most common greenhouse heating system [1,2].

Greenhouse heating is essential, even in areas with mild temperate climates, like the Mediterranean region, in order to maximize crop production in terms of quantity and quality and thus to increase the overall greenhouse efficiency [2]. Air heaters are used for heating greenhouse air either alone or in combination with heating pipes [2]. This combination can increase temperatures by 5 °C compared to using heating pipes alone, but it also implies a 19% increase in energy consumption [1] and a more heterogeneous climate distribution [2].

In southeastern Spain at cold times of the year farmers close the greenhouse ventilators and use thermal screens when these are available. Very few greenhouses are fitted with heating systems [5], usually due to the growers' reluctance to take on the outlay and running costs. The present work assesses the use of air heaters since the initial outlay is less than would be required for a system of water heaters and piping. It should be noted that air heaters are often the primary heating source in greenhouses in the Mediterranean area, where the heating needs are low [1]. Generally, hot-air heating is applied only during the night. The hot air is distributed to the crop via perforated polyethylene distribution ducts placed on the ground between the rows of plants [6]. The main advantage of air heaters is their prompt response to control changes in temperature, while the main disadvantage is the additional consumption of electricity [1] or fuel. A further drawback is that with this system the crop is cooler than the inside air, which may lead to condensation on the leaves if the dew point is reached; whereas, with pipe heating the crop is generally warmer than the air [4].

Studies on greenhouse heating systems usually focus on analysis of the greenhouse microclimate using a variety of air temperature and humidity sensors [7–11]. For example Perdigones *et al.* [12] evaluated two different heating systems (air heaters and heated flood) in a 6.6 × 20 m^2^ greenhouse in Madrid (Spain), with one sensor for outside temperature, one sensor for inside air temperature, and three for soil temperature. The present work proposes a methodology for studying both the horizontal temperature distribution and the airflow in a greenhouse equipped with two air heaters, using ten 2D ultrasonic anemometers, two 3D ultrasonic anemometers and a thermographic camera, as well as air temperature and humidity sensors. This methodology is similar to the proposal of Tadj *et al.* [2], who used a single 3D ultrasonic anemometer to measure air temperature and speed inside a greenhouse. Using a commercial Computational Fluid Dynamics (CFD) code, these authors evaluated experimentally and numerically the influence of three different heating systems on greenhouse microclimate (heating pipes alone, air heater alone, and a combination of the two). The experimental values were used to validate the simulation model.

As a first step to improve the design of the heating system using perforated polyethylene ducts, we have studied with sonic anemometry the airflow pattern and the temperature distribution generated by the systems normally used in Almería and in other Mediterranean countries [13]. It is important to study the airflow pattern generated by the heater fan and the distribution duct to analyze the heat transfers by forced convection inside greenhouses using this type of heating systems. Knowledge of the temperature distribution could help to improve microclimate homogeneity by modifying the design or improving the control of these heating systems [2]. Though these systems are not in widespread use in the Mediterranean area, and particularly in Almería, technicians need to know how the different heating systems work in order to improve them and adapt them to the requirements of the local climate and economy. The methodology presented here is adapted from that of a previous work in which two 3D ultrasonic anemometers and six 2D ultrasonic anemometers were used to measure natural ventilation in greenhouses [14]. These devices have been adapted to carry out measurements inside the greenhouse, focusing on air temperature distribution.

## 2. Experimental Setup

### 2.1. Site and Greenhouse Description

**Figure 1.**
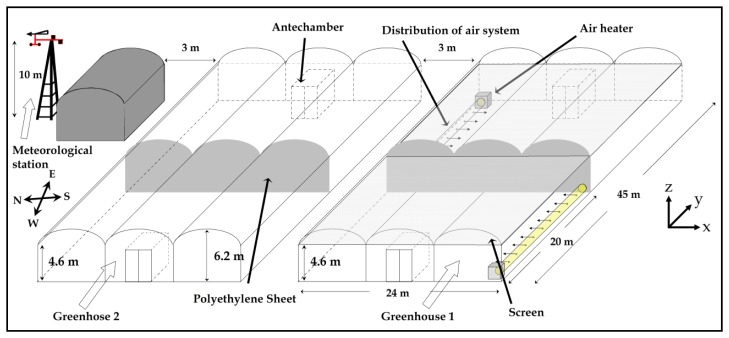
Location of the experimental greenhouse at the farm.

The experimental work took place in two multi-span greenhouses (24 × 45 m^2^) located at the agricultural research farm belonging to the University of Almería (Figure 1), in southeastern Spain (36°51′N, 2°16′W and 87 m elevation). The experimental greenhouses were physically divided into two similar sectors by a polyethylene sheet fixed to a stainless steel structure. The measurement tests were carried out in the eastern halves of the experimental greenhouses (24 × 25 m^2^).

The crop analyzed was *Cucurbita pepo* L. cv. Canella sown directly in the greenhouse soil. The crop was grown over the spring-summer cycle (February to June of 2011) in a sowing design of pairs of parallel lines 1 m apart, with 1.50 m between pairs of lines and 1 m between plants.

Greenhouse 1 was equipped with two air heaters and polyethylene distribution ducts. While the experiments heaters were operating, the ventilators were closed and an Aluminet 50-I aluminized screen (Polysack Plastic Industries, Ltd., Nir Yitzhak, Israel; 50% to 54% shading) was in place in greenhouse 1. Among passive heating systems, movable thermal screens are one of the most practical and appropriate means for reducing heating requirements in greenhouses during nighttime in the winter months [8,15–17]. Thermal screens influence convection, radiation, and latent heat transfer [18,19]. Their principal effect is to provide additional thermal resistance that reduces the overall rate of heat transfer to the surroundings [16,17]. Teitel *et al.* [6] observed that a horizontal 20%-aluminized thermal screen automatically deployed every night kept the canopy temperature slightly higher than without a screen. The use of a thermal screen (acrylpolypropylene) increased plant temperature by 2 °C at night [18]. This type of screen was very efficient in reducing convective losses (by 50% or more), but less efficient for radiative losses [18]. However, the use of aluminized thermal screens also reduces the radiative losses, increasing the net radiation absorbed by the plants by about 100%, as a consequence of the reflection of long-wave radiation by the thermal screen [8]. Aluminized screens allow energy savings of between 15% [8] and 60% [20] and increases of 3 °C in canopy temperature [8]. The use of thermal screens during the night can reduce heat loss rates by 23–24% in plastic greenhouses [15] and the heating requirements by as much as 60 to 80% in a double-glazed greenhouse [16].

Greenhouse 2 (control) had neither heating nor aluminized screen and the ventilators were not operating during experiments. Tests were carried out before dawn and lasted approximately 2 hours (Table 1), during this time the heating system was always operating.

Although the two experimental greenhouses were equipped with two interior fans that could increase the mixing of the inside air [21], the experimental measurement tests were carried out with these fans switched off with a view to only analyzing the airflow pattern generated by the polyethylene ducts.

### 2.2. Air Heaters and Distribution Ducts

**Figure 2.**
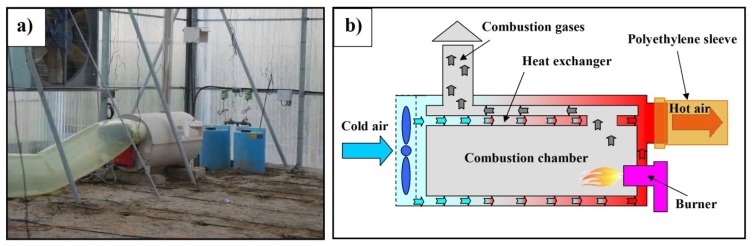
(**a**) Heater installed at the northeast corner of greenhouse 1. (**b**) Heater parts diagram.

In greenhouse 1, two Munters GP 80 heaters (Munters AB, Kista, Sweden) were installed, one at the northeast corner and one at the southwest corner (Figure 1). The air heaters (Figure 2(a)) work on the principle of indirect combustion, using a heat exchanger to separate the combustion process from the heated air stream, which allows combustion gases to exit the greenhouse through a chimney (Figure 2(b)). An axial fan, generating a volumetric airflow of 5,800 m^3^ h^−1^, forces greenhouse air at high pressure over the heat exchanger where it is heated with energy supplied by the combustion process. The heaters were equipped with a RG2 Gulliver burner (Riello S.p.A., San Pietro di Legnago, Italy) using light-oil, with a maximum consumption of 8.3 kg h^−1^ supplying a net output of 88 kW (for a maximum efficiency of 88.5%).

**Figure 3.**
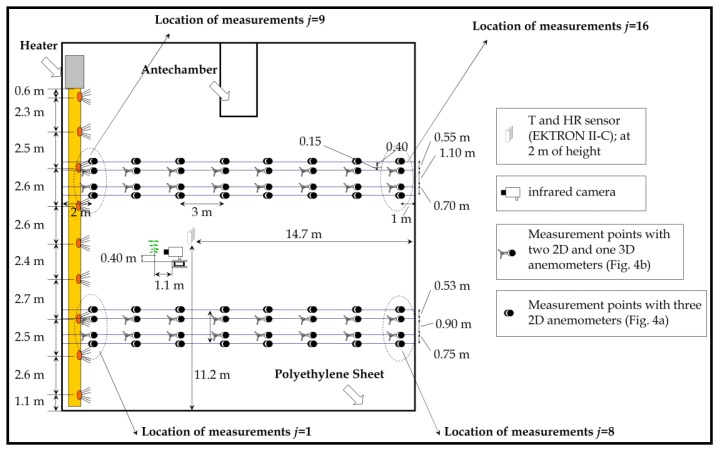
Measurement points with ultrasonic anemometers inside greenhouse 1.

The warm dry air is distributed throughout the greenhouse through a perforated polyethylene duct (75 cm diameter and 21.9 m long) located between the crops and the side wall (Figures 2 and 3). Each polyethylene duct has nine elliptical holes (horizontal axis 14 cm; vertical axis 11 cm) located 22 cm above the ground, in the lower part of the duct. The holes were handmade by a worker who made D-shape cuts in the polyethylene duct; this allows the plastic D to be bent outwards and deflect the discharged air jet downwards, perpendicular to the duct [13], thus avoiding direct collision with the plants. This design is recommended by the manufacturer and is the most common method employed in greenhouses fitted with this heating system in Almería.

### 2.3. Equipment and Instrumentation

Air velocity and temperature inside greenhouse 1 were measured with two 3D ultrasonic anemometers (mod. CSAT3, Campbell Scientific Spain S.L., Barcelona, Spain; resolution 0.001 m·s^−1^ and 0.002 °C; accuracy ±0.04 m·s^−1^ and ±0.026 °C). Air velocity was also measured with ten 2D ultrasonic anemometers (mod. Windsonic, Gill Instrument LTD, Lymington, United Kingdom; resolution 0.01 m·s^−1^; accuracy ±2%). Data were recorded by two microloggers (model CR3000, Campbell Scientific Spain S.L.), with a data registration frequency of 10 Hz for 3D sonic anemometers [22] and 1 Hz for 2D sonic anemometers [14].

**Figure 4.**
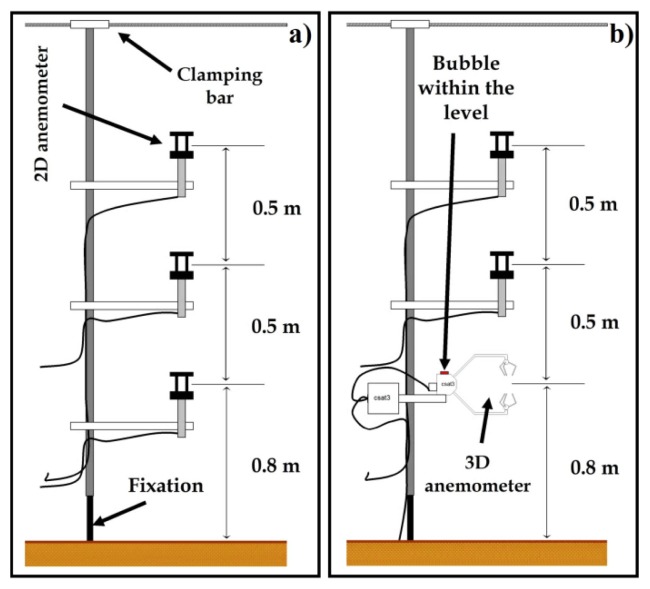
Details of the experimental setup using three 2D ultrasonic anemometers (**a**) and (**b**) one 3D and two 2D ultrasonic anemometers.

Figure 3 shows the locations of the sixteen (from *j* = 1 to *j* = 16) airflow measurements taken in the eastern sector of greenhouse 1. Air speed in the greenhouse was measured at each location using two devices equipped with three 2D ultrasonic anemometers (Figure 4(a)) and two devices equipped with one 3D and two 2D (Figure 4(b)) ultrasonic anemometers. The four devices were moving simultaneously and placed at each of the sixteen locations (Figure 3); recordings were taken at three different heights, providing a total of 192 measurement points. This number of measurement points (192) was greater than the 57 points used in a similar work [2]. At each of the sixteen anemometer locations (from *j* = 1 to *j* = 16, Figure 3) we measured air speed over 3 min [14]; this time period is a compromise between a shorter one which may reduce accuracy and a longer one which may increase the overall differences with regard to outside microclimate parameters [14,23]. Ultrasonic anemometers are able to determine the air velocity vector and the sonic temperature. The tests took 2 hours on average, generating a considerable amount of data. Two MATLAB 7.0 programmes were designed to process the air velocity data, one each for measurements taken by the 2D and 3D ultrasonic anemometers. These programmes allow us to analyze a complete experiment in a matter of a few minutes [14].

Outside climatic conditions were recorded by a meteorological station placed at a height of 10 m and located to the north of the greenhouse (Figure 1). The meteorological station included a BUTRON II (Hortimax S.L., Almería, Spain) measurement box equipped with a temperature sensor (Pt1000 IEC 751 class B, Vaisala Oyj, Helsinki, Finland) with a measurement range of −10 °C to 60 °C and an accuracy of ±0.6 °C. This measurement box was also equipped with a capacitive humidity sensor (HUMICAP 180R, Vaisala Oyj) with a measurement range of 0% to 100% and an accuracy of ±3%. Outside wind speed was measured with a Meteostation II (Hortimax S.L.), incorporating a cup anemometer with a measurement range of 0 to 40 m·s^−1^, accuracy of ±5%, and resolution of 0.01 m·s^−1^. Wind direction was measured with a vane (accuracy ±5° and resolution 1°). Solar radiation was measured using a Kipp Solari (Hortimax S.L.) sensor, with a measurement range of 0 to 2,000 W·m^−2^, accuracy of ±20 W·m^−2^, and resolution of 1 W·m^−2^.

Temperature and humidity inside the eastern sectors of the two greenhouses were measured with two EKTRON II-C measurement boxes (Hortimax S.L.) equipped with the same temperature and humidity sensors as the BUTRON II measurement box. These measurement boxes were placed in the middle of the eastern sectors of the two greenhouses at a height of 2.0 m.

To estimate air temperature (*T_S_*) from the speed of sound measured by the 3D ultrasonic anemometer, we must consider that the speed of sound in moist air depends on both temperature and humidity. From the inside humidity data recorded by the fixed sensors, we can obtain the specific humidity (*q*) and correct the sonic anemometer temperature (*T_SC_*, °C) using the following expression [24]:

(1) $$T_{sc}=\frac{T_{s}}{1+0.51q}$$

To improve the accuracy of this correction of the sonic temperature a humidity sensor would be required together with each ultrasonic anemometer, measuring the air humidity at the same points as the sonic temperature is measured.

To study the distribution of temperature difference between inside and outside the greenhouse Δ*T_io_* (*T_i_* measured by 3D anemometers at 0.8 m height) we need to consider the effect of changes in outside-inside temperature throughout duration of the tests. We used the difference in corrected air temperature *ΔT_io_^c^* [°C] with the average difference between inside-outside temperatures proposed in [21]:

(2) $$\Delta T_{io,j}^{c}(1)T_{sc,j}^{c}-T_{o}=T_{sc,j}\frac{T_{o}+T_{i}}{T_{o,j}+T_{i,j}}-T_{o}$$

where *T_sc,j_* is the corrected sonic temperature inside the greenhouse (Equation (1)) for position *j*, *T_o,j_* and *T_i,j_* are the mean outside and inside air temperatures recorded by the fixed sensors over the 3 minutes used for measurement at position *j*, and *T_o_* and *T_i_* are the mean outside and inside air temperatures during the test.

The crop temperature was measured with a compact infrared camera ThermoVisionTM A40-M (FLIR Systems AB, Danderyd, Sweden), with a spectral infrared range of 7.3–13 μm, a temperature range of −40 to +120 °C and an accuracy of ±2%. Emissivity of the upper side of leaves of *Cucurbita pepo* L. for the spectral infrared range of the infrared camera used in this work was determined at 0.985 [25]. One plant in the central area of the greenhouse was selected to be monitored by measuring its temperature with the thermographic camera every 5 min (Figure 3).

Air speed and temperature at the nine outlets in the polyethylene distribution duct connected to the air heaters, and at the very outlet of the air heater, were measured using a TESTO^®^ 445 multifunction digital handheld instrument (Testo S.A., Cabrils, Spain) with a hot bulb probe for the measurement of velocities in a range of 0 to 10 m·s^−1^, with an accuracy of ±0.03 m·s^−1^ and resolution of 0.01 m·s^−1^. The omni-directional hot bulb anemometer measures the magnitude of the speed vector. The equipment also contains a temperature probe (thermistor NTC) with a range of −20 to 70 °C and an accuracy of ±0.4 °C.

## 3. Results and Discussion

The outdoor climatic conditions during the three experiments are summarized in Table 1. Minimum outside temperature was 7.7 °C for experiment 1 and 9.3 °C for experiments 2 and 3. The minimum value inside the unheated greenhouse 2 was 10.4 °C, 9.2 °C and 10.0 °C for experiments 1, 2 and 3, respectively. These values, which are lower than the set heating temperature (12 °C), indicate that the climatic conditions were suitable to test the heating system. Although this is not the usual operational mode, the heaters were operated without interruption over the course of the experiments, since this allowed measurement of air speed at all the measurement points (Figure 3).

### 3.1. Airflow Characteristics

**Figure 5.**
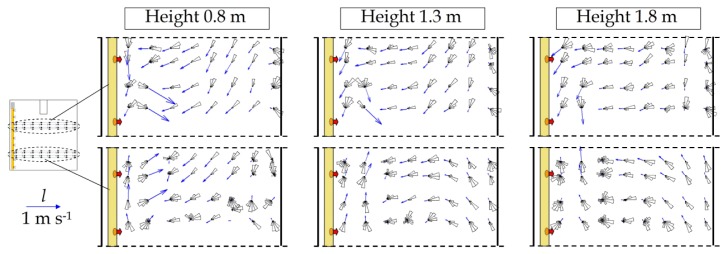
Two-dimensional resultants of air velocity in the *XY* plane (*l*) and polar plots of airflow direction in measurement test 1.

**Figure 6.**
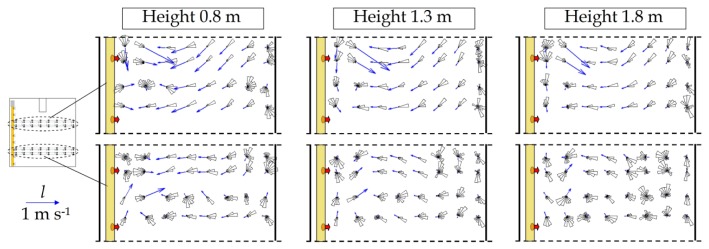
Two-dimensional resultants of air velocity in the *XY* (*l*) and polar plots of airflow direction in measurement test 2.

**Figure 7.**
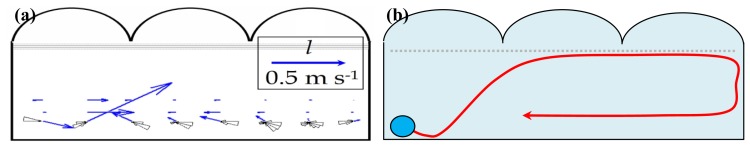
(**a**) Two-dimensional resultants of air velocity in the *XZ* plane (*v*) and polar plots of airflow direction in measurement test 2 (measurement line 2). (**b**) Approximate representation of the inside airflow.

The methodology proposed in the present work provides a detailed description of the forced airflow generated by the air heaters. Figures 5 and 6 show the two-dimensional resultant of air velocity in the *XY* plane (*l*) and the frequency histograms of velocity directions (depicted as polar plots) inside greenhouse 1. The vertical component was only measured with the 3D ultrasonic anemometers, and so we can obtain the two-dimensional resultant of air velocity in the *XZ* plane (*v*) for a height of 0.8 m (Figure 7(a)).

The horizontal component *l* increases as the measurement height decreases, with maximum values at 0.8 m (Table 2).

This is mainly due to the high airflow velocity on passing through the openings in the polyethylene distribution duct which distributes the forced warm air generated by the heaters (average values, measured with the omni-directional hot bulb anemometer, of 23.4, 22.9 and 31.8 m·s^−1^ for tests 1, 2 and 3, respectively). The variation in air speed at different heights was also reported in a similar work using one air heater at 2.6 m height. In that work the mean air velocity at crop level was 0.2 m·s^−1^, increasing to 0.5 m·s^−1^ near the greenhouse roof, but with the highest values of 3 m·s^−1^ close to the air heater [2].

The results obtained for the vertical component *v* indicate that the warm air leaving the openings in the polyethylene distribution duct, less dense than the mass of cold air surrounding the crop, rises due to buoyancy a few meters away from the openings. The openings are directed towards the ground, and so the warm air first rebounds off the soil before rising to the higher part of the greenhouse (Figure 7(a)). We perceive that this warm air rises above the crop, reaching the southern side of the greenhouse and then descending to crop level before heading northwards towards the polyethylene distribution duct once more, thus establishing a clockwise cycle (Figure 7(b)). Overall, greater fluctuation in air direction is observed in the southern sector, in the area of the greenhouse that is furthest from the heaters (Figures 5 and 6). Here the airflow passes along the highest part of the greenhouse towards the southern side, which it hits, then being obliged to change direction and flow towards the north of the greenhouse (Figure 7(b)).

The airflow diagram in Figure 7(b) is similar to that obtained by Tadj *et al.* [2] using CFD simulations, though in that case the heaters were placed in the upper part of the greenhouse (2.6 m). When the heaters alone are used warm air accumulates in the upper part of the greenhouse, giving rise to temperature differences between the warm upper part and cooler lower part of up to 10 °C in a greenhouse of 4.1 m height [2]. In our case, with the heaters and the polyethylene distribution duct placed at ground level, this vertical gradient of air temperature should be greater than the 10 °C observed with the heaters placed in the upper part of the greenhouse [2], although the measurements taken do not allow us to confirm this. The location of holes produced a heat air jets towards down that collide with the ground and is reflected up onto the greenhouse (Figure 7).

**Figure 8.**
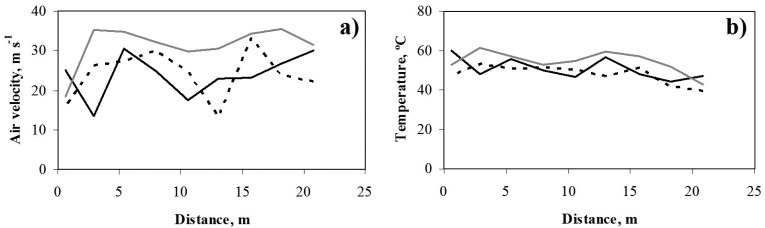
(**a**) Air velocity; (**b**) temperature distributions along the duct for the three tests carried out the 10/02/2011 (Img), the 16/02/2011 (- - -) and the 18/02/2011 (–––, black).

As the holes have been perforated manually in the polyethylene duct, the holes diameter and shape were heterogenic, and as consequence the air velocity through the different holes was non uniform (Figure 8(a)) and the heat supply change between the different holes. To get a uniform heat output along the duct it is necessary to increase the amount of air discharged along the duct [13] since the air temperature in the ducts falls continually (Figure 8(b)). The flow rate of air through the holes depends on the pressure difference between the inside the duct and outside. Therefore, holes diameters need to increase along the duct. An easy way to achieve good air distribution is to change the hole spacing [13] that in our case was uniform.

The aluminized screen is used to avoid warm air rising to the upper part of the greenhouse, away from the crop area. For this experiment the screen was placed at a height of 4.75 m, which does not prevent the warm air from rising above the crop. With a view to optimizing the heating system, we propose placing fans in the upper part of the greenhouse directed downwards to assist air circulation. Although this option has the disadvantage of involving extra energy consumption, the fans increase the air velocity and favor the mixing of air [21]. The optimal location and orientation of these fans must be determined in order to improve the temperature distribution inside heated greenhouses. The aluminized mesh should be placed at a lower height (3 m approximately), with a view to maintaining the warm air as close as possible to the crop; the use of such screens also helps to reduce the horizontal temperature gradient [26]. Another option is to add secondary polyethylene distribution ducts to distribute the warm air parallel to the crop lines [9]. This method improves the circulation of warm air in the area occupied by the crop, but on the other hand it hampers pruning, harvest and crop care. In fact, this kind of solution can realistically only be adopted in hydroponic crops where the cultivation gullies are placed over the ground and the polyethylene distribution ducts can be placed under them without hampering crop care.

### 3.2. Interior Microclimate

The temperature values reflected in Table 3 were measured in the lower part of the greenhouse (2.0 m height using the EKTRON II-C measurement box and 0.8 m height using the 3D ultrasonic anemometers). Analysis of the airflow inside the greenhouse revealed that the warm air generated by the heaters rises and flows above the crop until it reaches the southern side of the greenhouse. Thus, most of the heat accumulates in the upper part of the greenhouse [2]. Indeed, the temperature values presented in Table 3 could be much lower than the temperature reached in the upper part of the greenhouse; for example Tadj *et al.*[2] observed differences between the warm upper part and cooler lower part of up to 10 °C in a greenhouse of 4.1 m height; with similar outside conditions (outside temperature of 10.0 ± 1.1 °C and relative humidity of 65 ± 7%) the temperature near the greenhouse roof was 25.0 °C as opposed to 15.5 °C near the crop. A great amount of the heat generated by the heaters in these experiments does not go towards increasing the temperature of the air around the crop.

At a height of 2.0 m our heating system maintains the average temperature of greenhouse 1 (heated) 7.7, 8.8 and 7.3 °C above the temperature recorded in greenhouse 2 (unheated) in experiments 1, 2 and 3, respectively. Compared to outside conditions the heaters allow a temperature difference of 11.2, 8.2 and 7.6 °C, respectively. At 0.8 m the difference with outside temperatures was 11.1, 7.6 and 7.2 °C, respectively (Table 3). The small differences between temperatures at 0.8 and 2.0 m indicate that most of the heat moves to the upper part of the greenhouse.

As expected, these results are similar to those obtained by other authors. The maximum difference in air temperature between inside and outside the greenhouse 1 was 11.2 °C in test 1 (Table 3), which is slightly higher than the 10.2 °C recorded in a 6.6 × 20 m^2^ arched roof greenhouse in Madrid (Spain), in an experiment with four 9 kW electric air heaters [12]. With heating pipes, in a 6.5 × 31 m^2^ glass-covered greenhouse in Thessaly (Greece), Kittas *et al.* [8] observed an inside-outside difference in air temperature of 10.8 °C (with thermal screen) and 10.0 °C (without thermal screen). On the other hand, in a 8 × 20 m^2^ tunnel greenhouse in Thessaly (Greece) Bartzanas *et al.* [1] maintained an air temperature difference of 10 °C between inside and outside air with heating pipes alone, and of 15 °C when an airheater was also used.

Baille *et al.* [27] evaluated the night energy balance of an air-heated greenhouse in mild-winter climatic conditions in our region (Almería, Spain). These authors observed an average difference in inside-outside temperature of 7.6 °C in February for a low heating level (27 kW; 62 W·m^−2^) and of 14.1 °C in March for a high heating level (58 kW; 134 W·m^−2^). In the present experiment the heater was more powerful (88 kW; 146.67 W·m^−2^), and the maximum difference was 11.2 °C in test 1. This lower inside-outside temperature difference was possibly due to the greater volume of air in the experimental greenhouse (3,420 m^3^ in the eastern sector), as opposed to the Almería type greenhouse used by Baille *et al.* [27] (1,500 m^3^).

### 3.3. Horizontal Distribution of Inside Temperatures (at 0.8 m Height)

**Figure 9.**
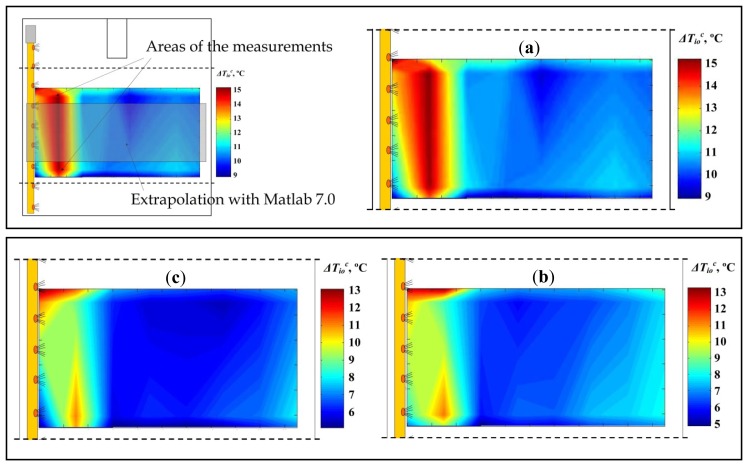
Difference in corrected air temperature (*ΔT_io_^c^*) in greenhouse 1 (heated) at 0.8 m height. Tests 1 (**a**), 2 (**b**) and 3 (**c**).

Using the difference in corrected air temperature *ΔT_io_^c^* [°C] with the average difference between inside-outside temperatures (Equation (2)), sonic anemometry allows us to obtain the temperature distribution maps for greenhouse 1 with the air-heaters (Figure 9), revealing a great degree of heterogeneity of inside temperature.

Maximum values are reached in the part of the greenhouse closest to the hot air outlet. The previous analysis of airflow showed that the warm air rises on leaving the polyethylene distribution duct and flows along the upper part of the greenhouse towards the southern side. When it reaches the southern side and descends to the ground it is much cooler than the maximum values recorded (Figure 9). Using this heating system the maximum temperature differences between the warmest and coolest points were 6.5 °C, 8.8 °C and 8.3 °C for experiments 1, 2 and 3, respectively. The coolest points were found in the central area of the greenhouse, not on the southern side as might have been expected. The temperature distribution maps show marked longitudinal temperature gradients, but slight transversal variation, which is typical for this warm air distribution system [28].

The considerable heterogeneity of temperatures may give rise to irregular crop development. This could be solved in three ways: (i) increasing the holes diameters along the duct or changing the holes spacing, (ii) placing the aluminized mesh at a lower height, if the crop allows, or (iii) improving the distribution of hot air inside the greenhouse, both of which were discussed in Section 3.1.

The use of a single air heater leads to greater climatic heterogeneity inside the greenhouse than the system of warm pipes or a combination of the two systems [2]. With a view to reducing this heterogeneity of the microclimate, different methods can be employed to distribute the warm air produced, for example using a network of tubing placed above the crop lines [9]. The results obtained indicate the need to continue working on developing new systems which facilitate the mixture of air inside the greenhouse and therefore contribute to homogenizing the inside microclimate.

The methodology presented in this work could be applied to study the microclimate in greenhouses equipped with warm water pipe heating systems. Using sonic anemometry would allow the analysis of airflow and temperature distribution inside the greenhouse as in the present work. In addition, thermography would also enable researchers to: (i) study the crop temperature distribution using suitable values of emissivity [25]; (ii) analyze the temperature distribution in the network of warm water pipes, for which it would also be necessary to determine accurately the emissivity of the pipes.

### 3.4. Inside Crop Temperature

The main crops in the Mediterranean region are vegetables adapted to warm climates. These species are mostly grown in the warm season and they are suited to mean air temperatures of 17 to 28 °C, with mean daily temperature limits of 12 °C minimum and 32 °C maximum [29]. Von Elsner *et al.* [30] quotes mean temperatures of 17 to 27 °C and upper and lower limits of 10 and 35 °C, respectively. These species cannot withstand the cold and suffer irreparable damage if subjected to freezing conditions. Persistence of temperatures below 10–12 °C over several days can affect their productivity [29].

The mean crop temperature over the three tests was over 17 °C (Table 4). The crop temperature was measured approximately 7 m from the northern side of the greenhouse; these values must be considered with caution, since the crop temperature was only measured for one plant. Given the wide range of inside temperatures (Figure 9), the plants located in the central and southern parts of the greenhouse were likely to be considerably cooler. Another drawback of this heating system lies in the fact that the crop temperature tends to be lower than the air temperature [2] (Tables 3 and 4). Teitel *et al.* [4] concluded that with air heating, the crop was cooler than the inside air, whereas with pipe heating the crop is generally warmer than the surrounding air. The use of the air heater increases the crop aerodynamic conductance, due to both higher temperature differences between crop and air and higher air velocity above the crop [1]. Whenever the leaf temperature is lower than the air temperature there is a risk that it will reach dew point and that condensation will occur on the leaves. Condensation at night is a major problem in greenhouses, since it enhances the development of diseases (e.g., botrytis and late blight) [6]. This problem can be reduced by using a thermal screen [6,8] or by combining heating pipes and air heater [2]. When air heaters are employed, special care must be taken with the set temperatures to ensure that the temperature of leaf surfaces is maintained above the dew point. This is an excellent way to prevent condensation and therefore helps to limit common plant diseases, particularly fungal ones, in the greenhouse [31].

For the three tests carried out, the heating system was calculated to produce an increase in mean air temperature of 0.12 °C·min^−1^. It is essential to know this value in order to program the system depending on the initial greenhouse temperature on the one hand and the desired temperature to be reached on the other. Regarding the crop temperature, the system achieved increases of 0.09, 0.05 and 0.06 °C·min^−1^ for tests 1, 2 and 3, respectively.

### 3.5. Fuel Consumption

The results obtained in the present study confirm that heating power of 146.67 W·m^−2^ (an 88 kW heater for 600 m^2^) is enough to avoid low temperatures in winter that are typical of the Mediterranean climate, thus avoiding losses due to frost damage. On the downside, this heating system may well prove too costly for the grower if required to work frequently. In theory the consumption of these heaters is 8.3 kg·h^−1^ (9.8 L·h^−1^), and over the approximately 2 h that the tests lasted the consumption of diesel was between 18 and 20 L per heater. For instance, during the month of February 2011 the heating system was operating automatically for a total of 2,245 min, which makes a total consumption of 365.4 L of diesel at the mean theoretical consumption of 9.8 L·h^−1^. Bearing in mind the total production of the greenhouse, this additional heating cost does not seem justified; for greenhouse 1 (heated) the final production obtained was 6.1 kg·m^−2^, while for the unheated greenhouse 2 total production was 6.3 kg·m^−2^, *i.e.*, there are no statistically significant differences. In this case the heating system was only operational during the first month of the crop cycle (from the date of seeding to the 7 March 2011).

The same system was also used in a second crop cycle, from October 2011 to Mars 2012, with a total fuel consumption of 2,525 L. The heating system allowed an increase of average inside temperature from 15.9 °C (in the non heated greenhouse) to 17.6 °C. This increase of temperature improved the tomato production from 5.0 kg·m^−2^ to 6.5 kg·m^−2^. However, the value of the increase of yield was of 0.86 €·m^–2^ (with an average price of tomato marketed of 0.61 €·kg^−1^), whereas the cost of the fuel was 2.41 €·m^−2^ (with an average price of fuel of 1.03 €·L^–1^). Under these economic conditions (low tomato price and high fuel price), the use of the air heating system analyzed in this work results in a 39% reduction of grower turnover.

If the farmer can afford the costs of heating, in the light of these results using the heating system could only be recommended as a safety measure to prevent loss of production due to frost, as happened in January 2005 when temperatures reached the lowest levels ever recorded in Almería; absolute minima of 0.1 °C were recorded in Almería city (Spain) [32] (AEMET, *Agencia Estatal de Meteorología*) and −3 °C in the “*Las Palmerillas*” experimental station located in *El Ejido* (Spain) [33], at the centre of the largest concentration of greenhouses in the province.

In order to reduce the running costs of this heating system many recommendations can be found in the literature, and these should be taken into account with a view to future research works: the use of double glazing [34] or different types of covering materials such as glass, PE, PVC, *etc.* [15,35], the insulation of side walls [34,36], the use of thermal screens [8,37,38] the development of zigzag covering [39] and the development of Fresnel lenses for the south-facing roof cover [40]. Other authors propose novel heating systems such as infrared (IR) radiative sources [10].

For the present study a 50% aluminized thermal screen was used, and in the future denser screens could be tested, as they have been proved to contribute to reducing heating energy consumption [8,41]; these screens reduce the heat losses in greenhouse [38] and avoid warm air rising to the upper part of the greenhouse, maintaining it in the area occupied by the crop (see Section 3.1). For example, thermal screens can reduce the heat consumption (for heating pipes) by about 17% in daytime and 11% at night [41], though this author did not indicate the characteristics of the screen. Kittas *et al.* [8] estimated that a 65% aluminized thermal screen can lead to energy savings (for heating pipes) of about 15% during the winter period; in addition, the thermal screen provided a more homogeneous microclimate and increased both the average air temperature and the canopy temperature [6], while at the same time reducing the vertical gradient of temperature and humidity.

## 4. Conclusions

The methodology proposed in the present work has allowed us to determine the airflow pattern generated by an air heater of indirect combustion equipped with a polyethylene duct with a row of holes of equal diameter, the most widely used heating system in Almería's greenhouses.

Sonic anemometry has also allowed us to analyze the temperature distribution inside the experimental greenhouse. The average inside and outside temperatures (measured with fixed sensors) were used as the parameter to scale the air temperatures measured with 3D ultrasonic anemometers (corrected with the specific air humidity recorded in the middle of the greenhouse) at different positions and different times.

The air heating system using perforated polyethylene ducts, which is the most widely employed system in Mediterranean greenhouses, is not suitable with the specific design analyzed in this work. The constant diameter and spacing of the holes produce a decrease in the heat supplied by the system as a result of the reduction in outflow temperature and airflow along the duct. The location of outflow holes in the lower half of the duct ensures that plant damage is avoided. However, the jet of warm air collides with the ground and rises toward the roof of the greenhouse. The warm air is first stopped and redirected to the opposite sidewall of the greenhouse by the thermal screen. The side wall then forces it to descend, and finally it is driven toward the side wall where the duct is located.

On the one hand, this forced convection generated by the axial fan and the polyethylene distribution duct gives rise to considerable heterogeneity of temperature inside the greenhouse, and on the other it does not prevent the hot air from rising to the top of the greenhouse as a consequence of the buoyancy effect, and so most of the energy used by the heaters does not go towards heating the air surrounding the crop.

To obtain a more uniform heat output along the duct, the holes diameter should be increased to offset both the pressure drop and the temperature decrease along the distribution duct. To reduce the vertical gradient of temperature and avoid heat raising to the top of the greenhouse a better orientation of the air outlets should be studied. Other options that may contribute to a better temperature distribution inside the heated greenhouse could be the use of fans placed in the upper part of the greenhouse and directed downwards to assist air circulation or adding transversal polyethylene ducts to distribute the warm air between the crop lines. However, these last two solutions would increase the cost of installing the heating system.

Overall the air heating system is able to maintain the inside greenhouse temperature at suitable levels, 7.2 to 11.2 °C above the outside temperature. Although the crop temperature was below that of the air, the heating system maintained the crop temperature between 17.6 and 19.9 °C, *i.e.*, above the minimum recommended value (10 °C). Due to the high cost of fuel and the low prices of tomato, the air heating system analyzed in this work would only be profitable in the particular conditions of Almería greenhouses as a system to prevent frost.

## Figures and Tables

**Table 1.** Outside climatic conditions. *u_o_*, wind speed [m·s^−1^]. θ, wind direction [°]; *RH_o_*, relative outside humidity [%]; *T_o_*, outside temperature [°C]. *R_g_*, solar radiation [W·m^−2^].

**Date**	**Test**	**Time**	***u****_o_*	***θ***	***HR****_o_*	***T****_o_*	***R****_o_*
10/02/2011	1	07:50–10:11	2.16 ± 0.96	80 ± 16	81 ± 3	9.5 ± 1.2	59 ± 48
16/02/2011	2	06:49–08:49	1.47 ± 0.57	301 ± 25	46 ± 4	10.5 ± 0.6	6 ± 12
18/02/2011	3	06:55–08:47	1.33 ± 0.69	144 ± 116	46 ± 4	10.6 ± 0.8	5 ± 11

**Table 2.** Inside average values of two-dimensional horizontal resultant of air velocity in the XY plane *l* [m·s^−1^] and the XZ plane *v* [m·s^−1^]; height [m].

**Date**	**Test**	***Height***	***l***	***v*** ^a^
10/02/2011	**1**	0.8	0.23 ± 0.16	0.18 ± 0.19
		1.3	0.18 ± 0.11	-
		1.8	0.14 ± 0.10	-

16/02/2011	**2**	0.8	0.23 ± 0.17	0.21 ± 0.16
		1.3	0.18 ± 0.17	-
		1.8	0.14 ± 0.14	-

18/02/2011	**3**	0.8	0.24 ± 0.12	0.22 ± 0.15
		1.3	0.18 ± 0.08	-
		1.8	0.13 ± 0.07	-

**Table 3.** Inside climatic conditions. At a height of 2.0 m measured with an EKTRON II-C measurement box: *T_i_*, inside temperature [°C]; Δ*T_io_*, inside-outside difference temperature [°C]. At a height of 0.8 m measured with 3D ultrasonic anemometers: *T_sc_^c^*, sonic air temperature corrected with the average inside-outside temperature difference; Δ*T_io_^c^*, inside-outside difference in corrected air temperature [°C]; *T_max_-T_min_*, difference in temperature between the hottest and coldest point [°C].

**Date**	**Test**	***Greenhouse***	***T****_i_*	***ΔT****_io_*	***T****_sc_^c^*	***ΔT****_io_^c^*	***T****_max_****-T****_min_*
10/02/2011	**1**	1 (heated)	20.7 ± 5.5	11.2	20.6 ± 1.8	11.1 ± 1.8	6.5
		2 (unheated)	12.9 ± 2.7	3.4	-	-	-

16/02/2011	**2**	1 (heated)	18.7 ± 2.6	8.2	18.1 ± 2.1	7.6 ± 2.1	8.8
		2 (unheated)	9.9 ± 0.6	–0.6	-	-	-

18/02/2011	**3**	1 (heated)	18.2 ± 2.9	7.6	17.8 ± 2.2	7.2 ± 2.2	8.3
		2 (unheated)	10.9 ± 0.7	0.3	-	-	-

**Table 4.** Crop temperature during the tests measured with the compact infrared camera [°C].

**Date**	**Test**	***Average***	***Min***.	***Max***.
10/02/2011	1	19.9 ± 3.7	13.7	26.1
16/02/2011	2	17.6 ± 1.6	14.5	19.9
18/02/2011	3	18.1 ± 2.1	14.2	21.3

## Appendix

**Notation**	
*l*	two-dimensional horizontal resultant of air velocity in the XY plane [m·s^−1^]
*Q*	specific humidity [g·g^−1^]
*R_g_*	solar radiation [W·m^−2^]
*RH*	relative humidity [%]
*T*	temperature [°C]
*u_o_*	wind speed [m·s^−1^]
*v*	two-dimensional horizontal resultant of air velocity in the XZ plane [m·s^−1^]
**Greek Letters**	
Δ	difference
θ	wind direction [°]
**Subscripts**	
*i*	inside
*j*	measurement point
*max*	maximum value
*min*	minimum value
*o*	outside
*s*	sonic
*sc*	sonic corrected
**Superscripts**	
*c*	corrected
